# Mortality Risk Factors for Middle East Respiratory Syndrome Outbreak, South Korea, 2015

**DOI:** 10.3201/eid2111.151231

**Published:** 2015-11

**Authors:** Maimuna S. Majumder, Sheryl A. Kluberg, Sumiko R. Mekaru, John S. Brownstein

**Affiliations:** Massachusetts Institute of Technology, Cambridge, Massachusetts, USA (M.S. Majumder);; Boston Children’s Hospital, Boston, Massachusetts, USA (M.S. Majumder, S.A. Kluberg, S.R. Mekaru, J.S. Brownstein);; Harvard Medical School, Boston (J.S. Brownstein)

**Keywords:** Middle East respiratory syndrome, MERS, MERS coronavirus, viruses, infections, mortality risk factors, disease outbreak, South Korea

## Abstract

As of July 15, 2015, the South Korean Ministry of Health and Welfare had reported 186 case-patients with Middle East respiratory syndrome in South Korea. For 159 case-patients with known outcomes and complete case histories, we found that older age and preexisting concurrent health conditions were risk factors for death.

The ongoing outbreak of Middle East respiratory syndrome (MERS) in South Korea is the largest outside Saudi Arabia. As of July 15, the South Korean Ministry of Health and Welfare (MOHW) has reported 186 case-patients (*1*). Of these case-patients, 131 have recovered, 19 remained are hospitalized, and 36 had died (Figure 1). We conducted a preliminary mortality risk factor analysis for case-patients with MERS in South Korea who had known outcomes and covariates. We then compared our findings with those of previous investigations of case-patients in Saudi Arabia.

**Figure 1:**
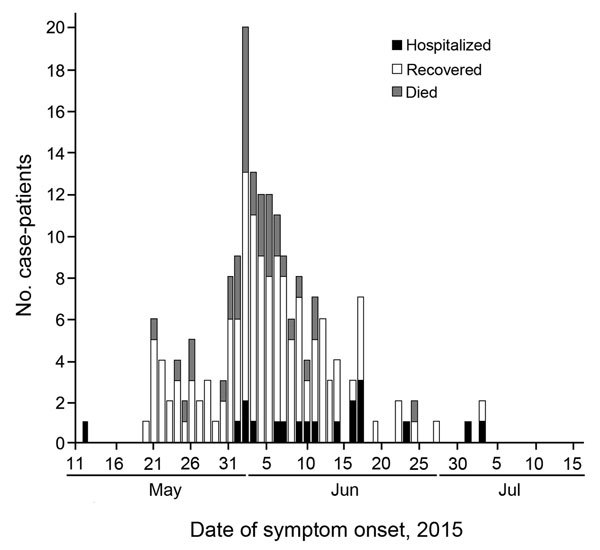
New case-patients with Middle East respiratory syndrome, South Korea, by date of symptom onset and patient status, as of July 15, 2015. When date of symptom onset was unavailable, date of reporting was used. Although all 186 reported case-patients are included in this plot, only case-patients with known outcomes (e.g., recovered, died) and dates of onset were included in the analyses (n = 159).

## The Study

Case identification numbers were matched between the June 26, 2015, World Health Organization (WHO) line list (*2*) and daily text-based MERS reports from the South Korean MOHW (*1*). Matching between the 2 data sources was conducted by using age, sex, and date of reporting. The WHO line list included additional risk factor data, which were cross-validated against meta data from the MOHW. The MOHW daily MERS reports provided real-time outcome information.

As of July 15, outcomes and covariates were publicly available for 159 of 186 case-patients, all of whom became ill during weeks 2–7 of the outbreak. We used this subset to describe the patient population, evaluate risk factors for death by using logistic regression models, and assess predictors of time from onset to diagnosis and onset to discharge by using Cox proportional hazards models. Five potential covariates were analyzed: sex, age, concurrent health condition status, health care worker status, and time from onset to diagnosis. For time-to-event analyses, patients were categorized into outbreak weeks by date of onset. We tested the Cox proportional hazards assumption by using Schoenfeld residuals and included an interaction term for predictor and follow-up time.

Of the 159 case-patients analyzed, 94 (59%) were men. Per WHO definitions, 25 (16%) had concurrent health conditions and 22 (14%) were health care workers. Age was normally distributed (range 16–87 years, mean [SD] 55 [15.9] years). All deaths occurred in patients >48 years of age. Time from onset to diagnosis was positively skewed: median 4 days (interquartile range [IQR] 2–7 days). Median time from diagnosis to death and from diagnosis to discharge were 13 (IQR 17–25.3) and 22 (IQR 9–16.5) days, respectively.

As of July 15, a total of 35/159 cases analyzed were considered fatal, which yielded an estimated case-fatality rate (CFR) of 22%. Univariate logistic regression models for each risk factor showed that older age and having a concurrent health condition were associated with death (both p<0.001); both variables remained significant after we adjusted for all 5 variables in a multivariate logistic regression model (Table). The model estimated that odds of dying were 7 times higher for persons with concurrent health conditions than for persons without these conditions (odds ratio 7.14, 95% CI 2.27–22.41). Furthermore, for every 1-year increase in age, odds of dying increased by 12% (odds ratio 1.12, 95% CI 1.07–1.17).

**Table:** Multivariate logistic regression model assessing odds ratios of risk for death for 159 case-patients with Middle East respiratory syndrome and known outcomes and covariates, South Korea*

Variable	Value	Odds ratio (95% CI)	p value
Male sex, no. (%)	94 (59)	2.85 (0.98–8.20)	0.052
Mean (SD) age, y	55 (15.9)	1.12 (1.07–1.17)	<0.001
Concurrent health condition, no. (%)	25 (16)	7.14 (2.27–22.41)	<0.001
Health care worker, no. (%)†	22 (14)	0.88 (0.09–8.93)	0.915
Median time-to-diagnosis, mo (IQR)	4 (2–7)	1.00 (0.89–1.14)	0.957

*IQR, interquartile range. †Only 1 health care worker died during the study period.

Time from onset to diagnosis decreased from a median of 10 days during outbreak week 2 (IQR 8.0–12.0 days) to 2 days during week 7 (IQR 1.0–2.0 days). There was a 43.7% average increase in hazard of diagnosis per week by a univariate Cox proportional hazards model (p<0.001). Separate univariate Cox models showed that no recorded risk factors were associated with this change.

Time from onset to discharge for patients who survived decreased from a median of 27 days during outbreak week 2 (IQR 22.0–32.0 days) to 19 days during week 7 (IQR 17.0–23.0). Univariate Cox proportional hazards analyses estimated a 34% average increase in the hazard of discharge per week (p<0.001), a 63% increase for health care workers (p = 0.046), and an 8% decrease for every 1-day increase in time-to-diagnosis. Multivariate analysis controlling for all risk factors showed that the increase in hazard by week decreased to 26% (p = 0.032); no other covariates remained significant.

## Conclusions

We found that older age and preexisting concurrent health conditions were associated with an increase in odds of death from MERS. Although being a health care worker appears to be protective, the association is not significant, probably because only 1 health care worker had died of MERS as of July 15, 2015. Time from onset to diagnosis was not an indicator for death, which suggests that the rapidity with which a patient receives supportive care may be of marginal consequence. Similarly, although being a male patient seems to increase odds of death, this relationship was not significant.

**Figure 2:**
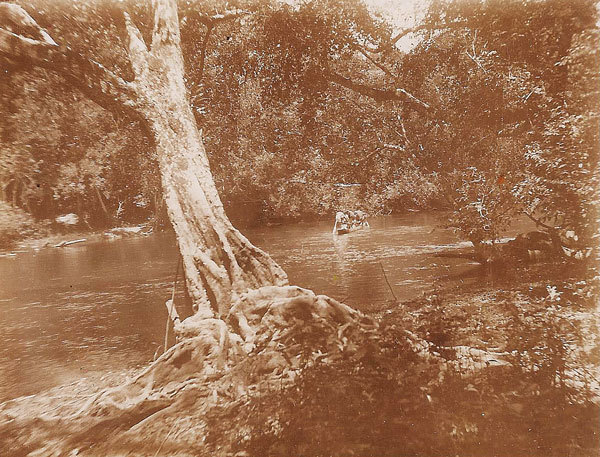
Cumulative proportion of case-patients with Middle East respiratory syndrome who were hospitalized, recovered, and died, South Korea, as of July 15, 2015. Total cumulative cases over time were calculated by date of symptom onset. When date of onset was unavailable, date of reporting was used. Cumulative recoveries and deaths over time were calculated by date of outcome; when date of outcome was unavailable, date of reporting was used. Although all 186 reported case-patients are included in this plot, only case-patients with known outcomes (e.g., recovered, died) and dates of onset were included in the analyses (n = 159).

On the basis of case-patients who had known outcomes through July 15, the ongoing MERS outbreak in South Korea had an estimated CFR (22%) that was half the CFR (44%) for all known case-patients with MERS in Saudi Arabia (*3*), but a CFR similar to that calculated for patients with only nonsporadic illness (21%) (*4*). Because 19 (10%) of 186 case-patients reported remain hospitalized, the final CFR of the outbreak might be higher than our current estimate. However, the proportion of patients who died (18%–19%) has been fairly stable since June 27 (Figure 2), which might indicate an asymptotic approach toward the final outbreak-specified CFR (*5**–**7*). If so, the final CFR associated with the MERS outbreak in South Korea during 2015 might be <22%. If all remaining hospitalized case-patients died, the final outbreak CFR would be 29%, which provides an upper limit for our current estimate of 22% excluding additional cases.

A total of 16 (64%) case-patients with MERS in South Korea who had concurrent health conditions died, compared with 19 (14%) case-patients without concurrent health conditions. This finding is comparable to that in a MERS study in Saudi Arabia, which reported a 60% CFR for a study population in which 45 (96%) patients had concurrent health conditions (*8*). Although only 25 (16%) case-patients had documented concurrent health conditions, the MERS outbreak in South Korean during 2015 has been largely nosocomial in nature. This finding suggests that observed differences between average CFRs in South Korea and Saudi Arabia might be driven in part by differential rates of concurrent health conditions for susceptible persons.

Use of publicly available data poses unique challenges. Although such data enable preliminary epidemiologic research during an ongoing outbreak, case information is stringently restricted to protect patient privacy. Because of this limitation, a follow-up analysis will be conducted pending availability of additional covariate data on potentially relevant biometrics (e.g., blood pressure) and behaviors (e.g., tobacco use), as well as outcomes for patients still hospitalized.

Despite these limitations, we found that risk factors for death among patients with MERS in South Korea who had known outcomes (age and concurrent health conditions) were similar to those identified for MERS case-patients in Saudi Arabia (*8**–**10*). Given these epidemiologic similarities and assuming that inherent virulence of MERS coronavirus is not context specific, the CFR difference might be caused not only by differential prevalence of risk factors but also by treatment or surveillance disparities.

Time to diagnosis decreased during the first 7 outbreak weeks, which probably contributed to the reduced length of hospitalization for patients who recovered and indicates that supportive care in South Korea might be highly adaptive. Furthermore, as reported by Cowling et al. (*11*), intensive case-finding activities might have produced more comprehensive diagnosis and reporting, thereby capturing less severe cases. In either event, given the frequency of importation events (*12*) and propensity for super-spreading (*13*), these findings provide information about MERS and MERS coronavirus in South Korea that might be useful in improving early case detection and preventing death.
